# Design and Evaluation of a Wearable Powered Foot Orthosis with Metatarsophalangeal Joint

**DOI:** 10.1155/2018/9289505

**Published:** 2018-09-19

**Authors:** Yixiang Liu, Xizhe Zang, Niansong Zhang, Ming Wu

**Affiliations:** ^1^State Key Laboratory of Robotics and System, Harbin Institute of Technology, Harbin 150080, China; ^2^Legs and Walking Lab, Shirley Ryan AbilityLab Formerly the Rehabilitation Institute of Chicago, Chicago, IL 60611, USA; ^3^Department of Physical Medicine and Rehabilitation, Northwestern University, Chicago, IL 60611, USA; ^4^School of Mechanical Engineering, Nanjing University of Science and Technology, Nanjing 210016, China

## Abstract

The metatarsophalangeal (MTP) joints play critical roles in human locomotion. Functional restriction or loss of MTP joints will lead to lower walking speed, poorer walking balance, and more consumed metabolic energy cost compared with normal walking. However, existing foot orthoses are focused on maintaining the movement of the ankle joint, without assisting the MTP joints. In this paper, in order to improve the walking performance of people with lower limb impairments, a wearable powered foot orthosis (WPFO) which has actuated MTP joint is designed and constructed. Preliminary experiments on three nondisabled subjects demonstrated functionality and capabilities of the WPFO to provide correctly timed dorsiflexion and plantar flexion assistance at the MTP joint during walking. These results also suggest that the WPFO could offer promise in certain rehabilitation applications and clinical treatment.

## 1. Introduction

Ankle-foot orthoses and prostheses have been studied widely over the past few decades for patients with walking disabilities caused by injuries or neurological and muscular pathologies such as trauma, stroke, cerebral palsy, spinal cord injuries, and muscular dystrophies. They are intended to enhance the basic walking function of patients by maintaining the range of motion of the ankle and foot [1] and providing stability during stance [2]. According to system actuation, these devices can be roughly divided into two categories, that is, passive devices and powered devices. Passive devices, as the name implies, do not have any active power sources and are not able to supply nonconservative positive work [3]. To improve their ankle push-off properties, the so-called energy storing and returning (ESR) mechanisms are proposed by the researchers [4]. Specifically, mechanical elements with elastic and damping characteristics are equipped on passive devices to harvest energy during loading and release it during push-off [5–7]. Contrary to passive devices, powered devices have onboard power sources and actuators that can provide positive and negative work to assist the users in the stance phase. They are usually actuated by electric motors [8–10], artificial pneumatic muscles [11, 12], linear or rotary pneumatic actuators [13–15], and hydraulic cylinders [16]. In these two categories of ankle-foot orthoses and prostheses, passive devices are relatively more popular in daily use due to their simplicity, compactness, durability, and comfort but have not been shown to sufficiently improve patients' biomechanics and energetic cost [17]. Nevertheless, some research has demonstrated that powered devices, although mostly at the research level, exert promise for improving the locomotor performance of patients, for instance, reducing walking metabolic energy [18] and achieving more natural gait [19].

One important issue existing in the research of powered ankle-foot orthoses and prostheses is that active assistance is applied only to the ankle joint. The metatarsophalangeal (MTP) joints are usually incorporated into the devices passively using an arc-shaped carbon-composite forefoot or a passive rotary joint [20–23] to mimic the rocker-like function of the forefoot [24]. This is mainly because the ankle joint produces substantially more work than other joints of the lower limb, in a burst late in the push-off phase [25]. However, the MTP joints actually have the largest range of motion among the other joints of the foot except for the ankle joint [26] and play important roles in human locomotion. Functional restriction or loss of MTP joints leads to lower walking speed, smaller step length, and more consumed metabolic energy compared with normal walking [27]. These facts indicate that to further improve the walking function of patients, it is necessary and will be beneficial to add actuated MTP joints into ankle-foot orthoses and prostheses and restore the normal range of motion of the MTP joints.

Therefore, in this paper, we propose a wearable powered foot orthosis (WPFO) that consists of an actuated MTP joint. The powered MTP joint is intended to provide assistance in dorsiflexion and plantar flexion when the foot pushes off the ground during walking. In order to verify the functionality and performance of the WPFO, preliminary experiments on three nondisabled subjects are carried out.

The rest of this paper is organized as follows.  Section 2 introduces the fundamentals of human walking gait and the MTP joints.  Section 3 presents the design of the WPFO device including mechanical structure and control system. The experiment procedure, data analysis, and results are presented in Section 4. Finally, the discussion and conclusion are given in Section 5 and Section 6, respectively.

## 2. Fundamentals of Human Gait and the MTP Joints

### 2.1. Human Gait

**Figure 1:**
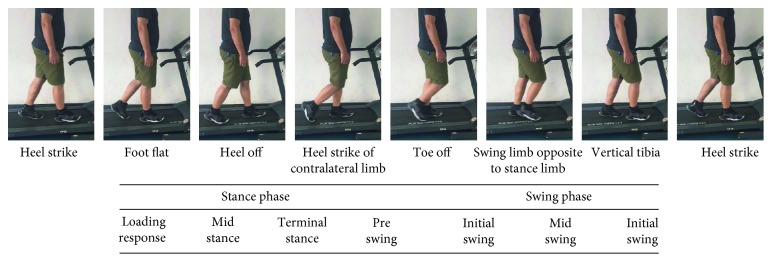
A gait cycle of human walking.

Human walking is the repetition of consecutive gait cycles in which the body is alternately supported by the right and left leg. A normal gait cycle presented in Figure 1 starts at the heel strike of one foot and ends at the next heel strike of the same foot. According to the contact of one leg and the ground in walking, a gait cycle can be divided into two phases, i.e., stance phase where the foot is in contact with the ground and swing phase where the foot is in the air. The stance phase ensures body progression while maintaining an upright posture, whereas the swing phase advances the leg to prepare for the next step [28]. Duration is about 60% of the gait cycle for the stance phase and the remaining 40% for the swing phase. The stance phase and swing phase can be further divided into multiple subphases including loading response, midstance, terminal stance, preswing, initial swing, midswing, and terminal swing. Each subphase with corresponding kinematic events and temporal distribution is defined according to the convention, as presented schematically.

### 2.2. The MTP Joints

During walking, the movement of MTP joints mainly occurs in the sagittal plane. In the terminal stance subphase, the heel lifts up from the ground and the body rotates relative to the toes around the MTP joints; in other words, the MTP joints are dorsiflexed. After reaching the maximum dorsiflexion angle of about 30° in the middle of preswing subphase, the MTP joints are rapidly plantar flexed towards the neural position, as having been shown in Figure 1 [29]. Although the MTP joints generate only a small amount of energy during walking, their movement especially dorsiflexion plays important roles in helping the ankle-foot complex to bear body weight and generate necessary propulsion force for forward walking. In our previous study [30, 31], we investigated the kinetic responses of human bodies when unilateral MTP joints were constrained during walking. Gait analyses of 12 healthy subjects were performed in terms of joint kinetics in two conditions, that is, walking normally and walking with the MTP joints of unilateral foot constrained. Based on the results, it was found that the constraints of the MTP joints had the most significant effects on the ankle joint. In more detail, the vertical and anterior/posterior (A/P) GRF, corresponding support and propulsion impulses, and moment and power of the ankle joint of the constrained leg significantly decreased. And it was more obvious when walking at a fast speed. This was perhaps owing to the protective mechanism of the human body. The constrained foot, of which the rotation around the MTP joint was affected by the constraints, was unsuitable for load bearing and propulsion.

## 3. Design of the WPFO

### 3.1. Mechanical Structure

**Figure 2:**
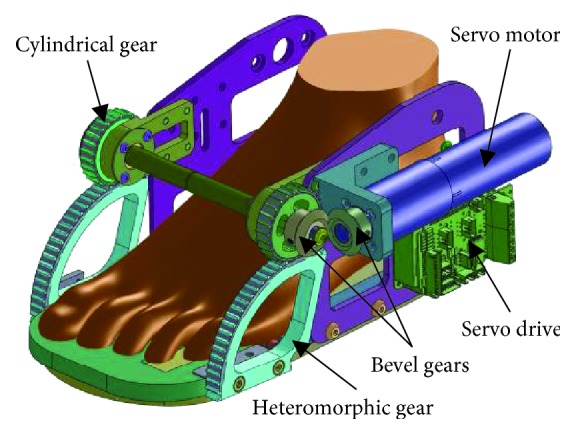
The virtual model of the WPFO.

**Figure 3:**
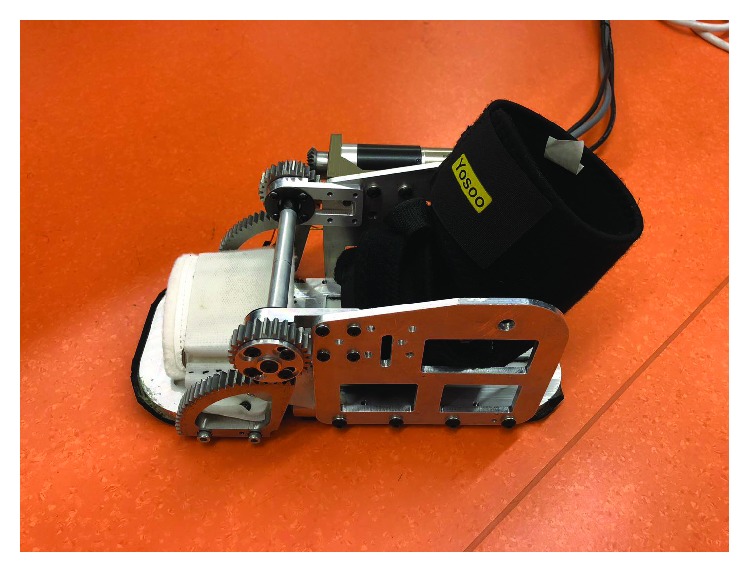
The prototype of the WPFO.

The virtual model and prototype of the WPFO device are shown in Figures 2 and 3, respectively. The sole of the WPFO consists of two parts, namely, the forefoot plate and the heel plate, which are hinged through a pivot shaft. The heteromorphic gears are connected with the forefoot plate, while the cylindrical gears engaged with the heteromorphic gears are connected with the heel plate. The servo motor (Maxon Motor AG, Sachseln, Switzerland) and servo drive (Elmo Motion Control Ltd., Petah Tikva, Israel) are mounted on the side plate which is connected to the heel plate. The cylindrical gear is actuated by the servo motor through a pair of engaged bevel gears. A layer of soft material is attached beneath the sole to soften the impact as the WPFO makes contact with the ground. The WPFO can be tied to the wearer's foot and shank through the braces. The total length of the WPFO is 270 mm, with distribution between the forefoot plate and heel plate in a similar ratio with the human foot. And its gross weight is about 1.2 kg. It should be noted that the weight can be reduced if locating the motor at a higher position, for example, on the leg or waist, and transmitting the actuation through some methods like rope dive. However, in this study, the motor is mounted on the foot to simplify the mechanical structure of the WPFO.

### 3.2. Control System

The control system hardware of the WPFO is composed of a controller, a servo drive, and two contact switches. For the sake of simplicity, a personal computer instead of a single-board microcontroller is adopted as the controller. One of the two contact switches is installed at the back of the heel plate, and the other one is installed on the forefoot plate near the pivot shaft. The signals of the contact switches are fed to the servo drive. The controller receives the feedback from the servo drive and sends commands to the drive based on the sensor status. The controller communicates with the drive via RS-232 protocol.

**Figure 4:**
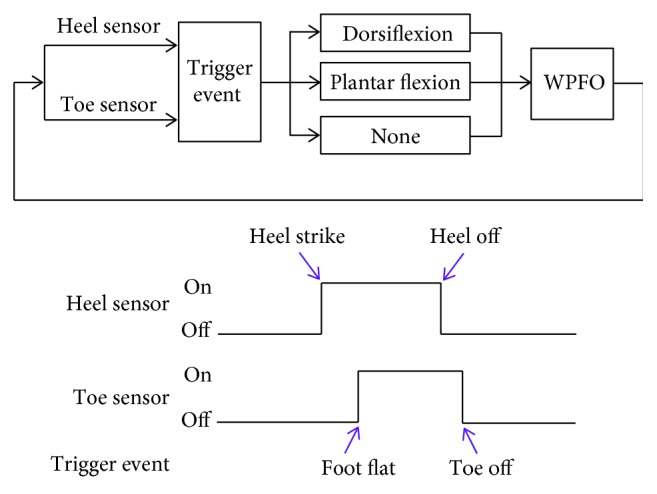
Working mechanism of the control system of the WPFO.

**Figure 5:**
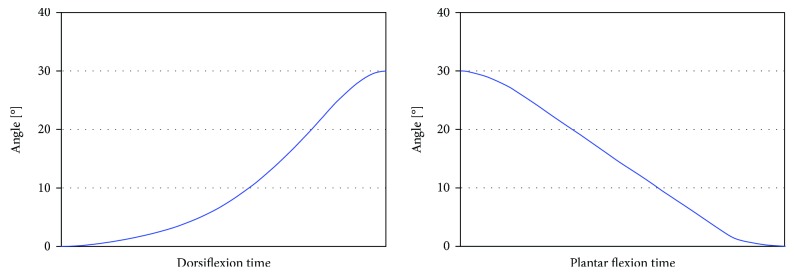
Reference trajectory of the MTP joint of the WPFO.

The control scheme of the WPFO is based on finite-state machine control, as illustrated in Figure 4. Gait events are detected when the switches make contact or lose contact with the ground. Specifically, when the heel lifts off from the ground (the heel sensor changes from on to off), the WPFO is triggered to provide assistive dorsiflexion torque until the toe starts to lift up; and when the toe lifts off from the ground (the toe sensor changes from on to off), the WPFO is triggered to provide assistive plantar flexion torque until the toe is fully off the ground. The lower-level motor controller is based on position tracking control. The reference trajectory of the MTP joint presented in Figure 5 is generated by a polynomial fit of human MTP joint kinematic data. At the start and the end of the trajectory, there are an accelerating segment and a decelerating segment, respectively, which are used to smooth the whole trajectory.

## 4. Experiments

### 4.1. Experiment Procedure

Three nondisabled subjects (mean ± standard deviation age 27 ± 3 yr, height 172 ± 8 cm, and weight 65 ± 12 kg) were recruited from Northwestern University to participate in the experiments. These subjects were experienced treadmill walkers and had no walking-related injuries such as muscle strains, joint sprains, or back injuries at the time of testing. And they all wore size 8-9 shoes in order to fit with the WPFO. All the subjects received written and verbal information about the experiment procedure and gave written informed consent prior to participation. The experiments were carried out in the Legs and Walking Lab of Shirley Ryan AbilityLab after the approval was obtained from the Institutional Review Board of Northwestern University.

In order to verify the functionality of the WPFO, subjects walked on a split-belt treadmill with embedded force plates (Fully Instrumented Treadmill, Bertec Corporation, Columbus, OH, USA) wearing the WPFO on the right foot. All subjects were instructed to walk at their self-selected speed under two conditions, that is, (1) without assistance torque and (2) with assistance torque. The order of experimental conditions was randomized across subjects. Under each condition, subjects completed 3 walking trials and walked for 2 minutes in each trial with a 2 min standing break inserted between two trials to avoid fatigue effect. Before the start of formal experiments, multiple practice trials were performed for subjects to acclimate to the devices.

### 4.2. Data Acquisition and Analysis

During the walking trials, ground reaction forces (GRF) on the right foot were collected by the force plates at 1000 Hz. And three-dimensional motion of the lower limbs was captured by an 8-camera motion capture system (NaturalPoint Inc., Corvallis, OR, USA) at 100 Hz. To make them available for analysis and comparison, the collected data were processed using custom programs written in MATLAB (The Mathworks, Natick, MA, USA) in the following method. GRF and marker position data were first low-pass filtered using a fourth-order Butterworth filter with cutoff frequencies of 20 Hz and 6 Hz, respectively [32], then were segmented into step cycles from heel strike to the next heel strike of the same foot. Because of the variability in the duration of each step cycle, the data were interpolated and resampled and then averaged across the last consecutive 20 strides to create a mean pattern [33]. After that, the obtained mean GRF of each subject were normalized to the corresponding body weight. In the end, the parameters of each subject were averaged across the 3 trials and further averaged across all subjects.

Based on the introduction in Section 2.2, the data including GRF, corresponding support and propulsion impulse, and ankle joint moment and power of the constrained leg were analyzed to evaluate whether the WPFO could restore the natural walking pattern. The support impulse was quantified as the time integral of vertical GRF during stance, and propulsion impulse was quantified as the time integral of A/P GRF when the force was directed forward [34]. Inverse dynamic analyses were performed in the sagittal plane to compute the ankle joint moment [35]. The joint power was then calculated by multiplying joint moments with joint angular velocities. Positive power occurs when the joint moment is in the same direction with the resultant angular velocity, while negative power occurs in the opposite direction.

### 4.3. Experiment Results

**Figure 6:**
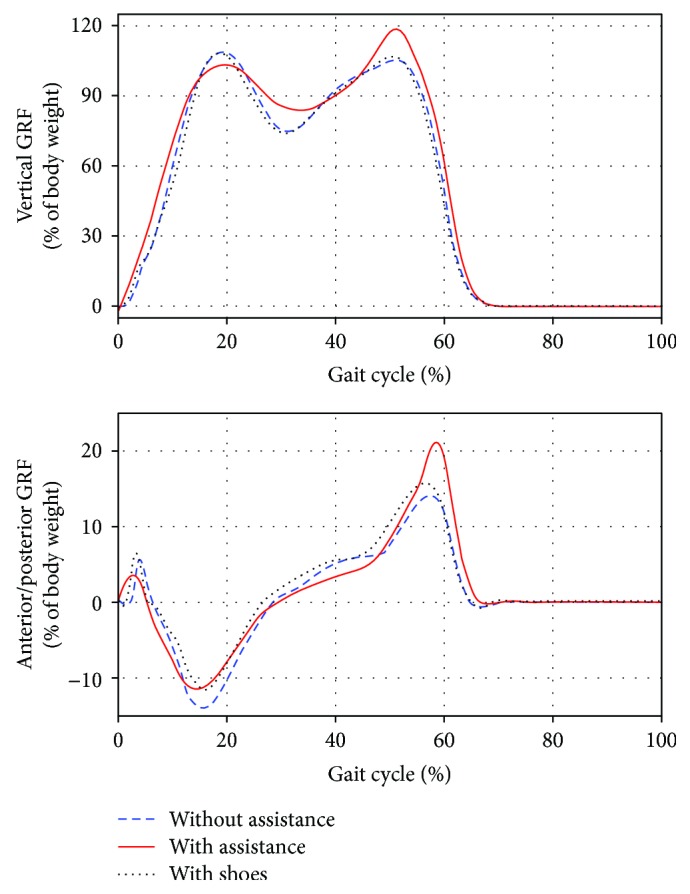
Averaged vertical GRF and anterior/posterior GRF during treadmill walking under the two conditions.

Figure 6 illustrates the averaged vertical GRF and A/P GRF acted on the right foot during treadmill walking under the two conditions. During the propulsive phase of walking, the peak value of support force (positive vertical GRF) increased by about 12% of the body weight, while the peak value of forward propulsive force (positive A/P GRF) increased by about 8% of the body weight.

**Figure 7:**
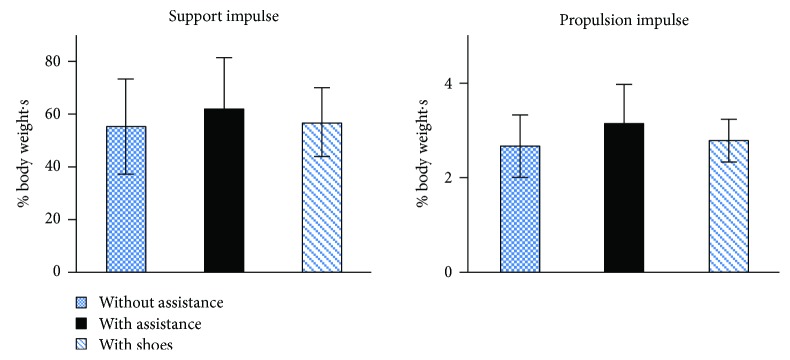
The support impulse and propulsion impulse during treadmill walking under the two conditions.

The support impulse and propulsion impulse incorporating both the magnitude and duration of vertical GRF and A/P GRF were analyzed and presented in Figure 7. Obviously, when walking with assistance torque, both the support impulse and propulsion impulse increased.

**Figure 8:**
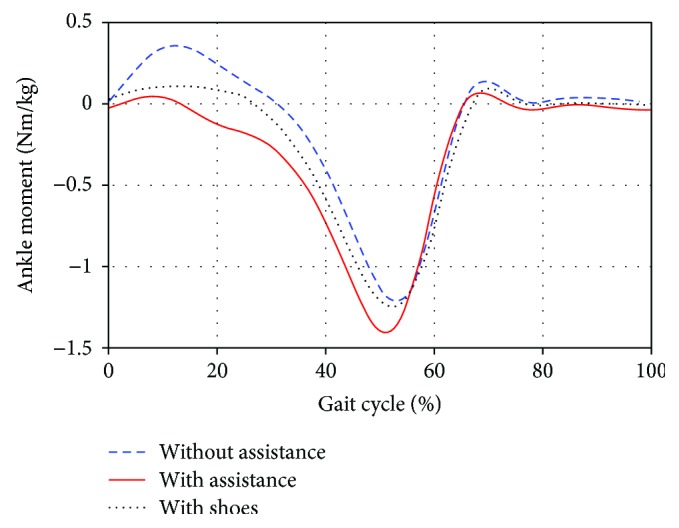
The right ankle moment over one complete stride for walking under the two conditions.

**Figure 9:**
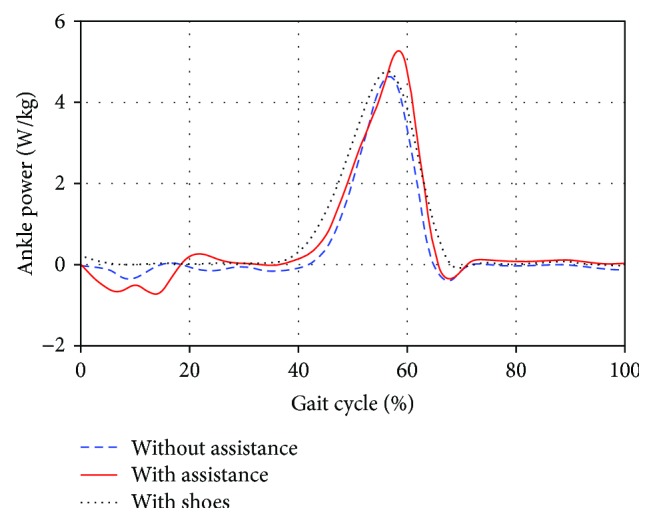
The right ankle power over one complete stride for walking under the two conditions.

Figures 8 and 9 present the moment and power of the right ankle over one complete stride of treadmill walking under the two conditions, respectively. The ankle moment for walking with assistance was larger than that without assistance. And increased peak power was generated by subjects at the push-off phase with the assistance of WPFO.

In the above figures from Figures 6to 9, the data of subjects walking with shoes are also presented. By comparing the data of walking without assistance and with shoes, it can be found that the WPFO without assistance had some small effects on normal gait patterns. Specifically, the GRF, impulse, and ankle joint moment and power slightly decreased. In addition, the comparison between the data of walking with assistance and without assistance showed that the WPFO with assistance allowed the gait pattern to return to a more normative behavior.

## 5. Discussion

The objective of this study was to design a novel wearable foot orthosis which had an actuated MTP joint to help patients to achieve dorsiflexion and plantar flexion of the MTP joint. From the experiments, we found that sufficient torque could be provided by a 200-watt electric servo motor that transmitted the power through a reducing mechanism. Pilot data collected from nondisabled subjects were used to demonstrate the function of the WPFO device. It was verified that the WPFO was able to provide both dorsiflexion and plantar flexion assistance during walking. The assistive capabilities of the proposed WPFO were most clearly illustrated by the vertical GRF and A/P GRF data during the walking trials with assistance torque. The increased second peak value of the vertical GRF and the increased peak of positive A/P GRF were indicative of larger push-off force that was provided by the WPFO for forward propulsion during the late stance phase. What is more, the timing of the GRF data and the moment and power of ankle joint showed small differences between the durations of stance phase in the two experimental cases, indicating that the WPFO could provide quick response and appropriately timed functional assistance.

Nowadays, plenty of people are suffering from lower limb disabilities caused by stroke, cerebral palsy, spinal cord injuries, or muscular dystrophies. The disabilities have severely affected their quality of life. Consequently, there is a growing demand for technological advances in orthotic and prosthetic systems, especially portable and powered devices. On the basis of our previous researches on the effects of the MTP joints on human walking performances, we proposed and constructed the novel WPFO that has a powered MTP joint. The WPFO provided a potentially new modality for applications in in-home assistive walking training and clinical rehabilitation or treatment. And hopefully, it may contribute to the improvement of the functional outcomes of daily training and treatment.

Although encouraging results are obtained from this study, there are still some important limitations to consider. Firstly, the WPFO device is a little bit heavy. To reduce the weight, the actuator and control system can be relocated on the leg or waist. Metal materials can also be replaced by composite materials with high strength and rigidity but low density. Secondly, the actuator adopted in this study is rigid. It will be better if compliant actuators like series elastic actuators or pneumatic artificial muscles are used, because inherent compliance is essential for human-machine interaction. Thirdly, the two contact switches can only detect the contact status between the ground and the two specific points on the sole. More force-sensitive resistors distributed beneath the sole can offer more reliable gait event detection during walking. Last but not the least, we only tested the WPFO on nondisabled subjects. More experiments have to be performed on patients with impairments to the MTP joints to further demonstrate the functionality of the device.

## 6. Conclusion

In this paper, the design and evaluation of a novel WPFO device with an actuated MTP joint are presented. The WPFO is proposed to help improve walking performance of patients with MTP joint impairments. The pilot data from three nondisabled subjects demonstrated the capabilities of the WPFO to provide functional assistance at the MTP joint during walking, which suggests that the WPFO could be potentially utilized in certain rehabilitation applications and clinical treatment. In the future, our research will be focused on the improvements to hardware structure and control scheme of the WPFO device.

## Data Availability

The data used to support the findings of this study are available from the corresponding author upon request.
